# Community involvement and engagement in designing a social-media based educational intervention for oral and sexual health promotion in transgender women in Malaysia: a narrative summary

**DOI:** 10.1186/s40900-025-00683-6

**Published:** 2025-03-27

**Authors:** Lahari A. Telang, Hezreen Shaik Daud, Zariq Rosita-Hanif, Betty Nizam, Aoife G. Cotter, Abdul Rashid

**Affiliations:** 1 RCSI-UCD Campus Malaysia (RUMC), Penang, Malaysia; 2 Centre for Experimental Host Pathogen Research (CEPHR), University College Dublin, Dublin, Ireland; 3 School of Medicine, University College Dublin, Dublin, Ireland; 4 Penang Family Health Development Association (FHDA), Penang, Malaysia; 5 Artist, Individual with lived experience, Penang, Malaysia

## Abstract

Patient and public involvement and engagement (PPIE) in health care research has been advocated to ensure that the research is relevant, acceptable and beneficial to the communities that it serves. However, there remains a significant gap in research projects incorporating PPIE among the transgender communities, particularly from the global South. In this narrative summary the authors describe the methodology of PPIE through a novel collaboration with community members for the design and development of a bilingual and culturally sensitive educational intervention that was tailor made for transgender women in Malaysia. The educational intervention named *Ms Radiance* was aimed at creating oral health awareness, improving oral health care utilization and promoting safe oral sex practices among transgender women. The PPIE team included local transgender community advocates and artists who played a pivotal role in making the educational content accessible, engaging and suitable for delivery through a popular social media platform, Instagram^®^. PPIE enhanced the intervention’s relevance and effectiveness through community friendly, culturally appropriate content creation, delivery and trust-building. Instructional designs such as the ADDIE (analyse, design, develop, implement and evaluate) ensure that educational interventions are well-planned and effective. A structed PPIE approach in the research project was seamlessly integrated with the ADDIE instructional design that guided the effective planning and delivery of oral health and sexual health education The research highlights the impact of PPIE on the project and offers insights for future interventions in marginalized communities from the global South.

**Supplementary Information:**

The online version contains supplementary material available at 10.1186/s40900-025-00683-6.

## Abstract (plain language summary)

Community involvement in healthcare research helps ensure that the work is meaningful, acceptable, and beneficial. However, research that includes transgender communities, especially in countries in the global South is lacking. This project focused on creating a bilingual and culturally appropriate educational program named *Ms Radiance* for transgender women in Malaysia. The program aimed to raise awareness about oral health, encourage using oral health services, and promote safer oral sex practices. To make sure the program was useful and relevant, researchers worked closely with transgender community members, including advocates and artists. They helped create educational materials that were easy to understand and engaging. These materials were shared on a popular social media platform, Instagram^®^, to reach the audience effectively. This paper aims to describe the involvement of the community in various stages of the project using a step-by-step process along with highlighting the benefits and challenges faced during the process.

**Supplementary Information:**

The online version contains supplementary material available at 10.1186/s40900-025-00683-6.

## Background

### Global disparities in transgender health care

The global health burden and health care needs of transgender people across the globe have garnered much interest in the last decade [1, 2] with most of the global trans-health literature being published in the last five years alone [3, 4]. However, only a minority of the literature on health burden and correlates demonstrate engagement with the trans-community [3]. The publishing sphere in transgender health research is dominated by literature from the global North, leaving significant gaps in literature from the global South [5, 6]. The phrase global South in the health care context has been used to describe countries that are lower in income compared to the more developed countries and often face political and cultural marginalization [7]. Gaps in transgender health literature from the global South are more pronounced from countries where identifying as transgender is criminalized [8, 9]. Transgender health research from the global South predominantly focus on HIV/STIs and/or sexual health and poorer health outcomes, with a dearth in reporting use of interventions and community based participatory research [5, 10]. Global literature regarding oral health care needs and service utilization among transgender and gender diverse populations is sparse [11]. The limited evidence from the global South indicates poorer oral health among gender diverse communities [12–14] and warrants wider studies with diverse representation of population for better understanding of oral health outcomes [11, 12]. The global strategy and action plan on oral health 2023–2030, laid out by the world health organization (WHO) emphasises the importance of implementation of evidence based, cost effective and sustainable interventions to promote oral health for all [15]. It is recommended that health care interventions planned for transgender people must be ethically grounded and focus on collaboration among community members, trans activists, scholars, and healthcare professionals [16, 17] through participatory research [18]. 

### Participatory research

Participatory research with transgender women stands to enhance local ownership of knowledge by prioritizing the voices of those within the community [18]. Patient and public involvement and engagement (PPIE) in health research refers to the involvement of participants including patients in the research process that can range from consultation and advisory roles to more integrated approaches [19, 20]. Incorporating public and patient perspectives based on their lived experiences ensure that the research is relevant, acceptable and beneficial to those it aims to serve [21, 22]. Even though the concept of participatory research has been recognized and advocated in research involving disadvantaged communities [23], it is not so evidently reported in research studies coming from the global South [24]. Sparse reporting may be attributed to inherent power imbalances with researchers being viewed as experts in the field who are often the drivers of the research and influence the direction it takes [25, 26]. Participatory research with transgender communities from the global South remains understudied [5] with even fewer studies focusing on oral health promotion [27]. While there is community engagement at the point of knowledge dissemination, participatory involvement at the outset to define objectives and all subsequent points of the project is rare, especially in studies from the global South [28, 29]. The novelty in participatory research lies in its capacity to improve representation in health research and promote health care policies that improve access and outcomes [3, 5]. Through this narrative the authors have described PPIE methodology with emphasis on the participation of transgender women in various aspects of the design and development of a novel digital health intervention to promote oral health and sexual health in relation to oral STIs. Their role in trust building, recruitment, content creation, delivery and feedback about the intervention is described.

### Digital health for transgender persons

Digital health interventions and mobile health (mhealth) resources have been shown to offer valuable information that can improve health care access to transgender and gender diverse populations globally [30, 31], and also in low- and middle-income countries [32]. However, majority of such digital interventions have focused on HIV/sexual health [31] with limited focus on other areas in health care such as oral health. Digital interventions focusing on oral health of transgender and gender diverse populations in LMICs from the global South remain underexplored. Malaysia is a developing country in South-East Asia that has achieved Universal Health Coverage for its citizens [33]. However, disadvantaged populations including transgender women face challenges in receiving health care [8, 34, 35]. Digital health interventions for transgender and gender diverse populations in Malaysia have shown promising scope [36–38]. However, there is a lack of culturally sensitive, tailor-made interventions and web-based sources which cater to the specific health care needs of transgender women locally [39, 40]. Being cognizant of the fact that involvement of stakeholders is of great value in digital health interventions it is essential to involve the community through co-production of ideas for engagement and effectiveness [41, 42]. In this report, the authors describe the involvement and engagement of transgender women in a novel collaboration for designing a social media-based educational intervention for their community. The overarching aim of the research was to create oral health awareness and improve oral health care utilization and safe oral sexual practices among transgender women in Malaysia. The combination of oral health promotion along with safe oral sexual practices was intentional to highlight the risk of STIs transmission through unprotected oral sexual practices. This project served as a valuable learning experience for the researchers as well as the members from the transgender community. The integration of and influence of PPIE on the study is described in this narrative. The authors prefer to refer to PPIE in this paper as: “participant” and public involvement and engagement in research as opposed to patient and public involvement and engagement [43]. 

## Methodology

### Ethics approval

Navigating the ethical challenges in conducting participatory research in marginalized communities within vulnerable contexts demanded broader discourse. Considerations to reflexive approach, cultural sensitivity, empathy and trust building were crucial elements to be incorporated [16, 44]. Hence the ethical approval for this project was detailed and meticulous. Due to the distinctive nature of the project, separate ethics committee approvals were sought in order to strengthen the ethical approach. First, from the local ethics committee where the study data was collected *(Joint Ethics Committee of School of Pharmaceutical Sciences*,* USM-Hospital Lam Wah Ee on Clinical Studies (USM-HLWE/IEC/2023 (0003))*, followed by a second ethics approval from the University that was adjudicating the PhD research *(Human research ethics committee of University College Dublin (UCD) Human Subject (Sciences) (LS-23-68-Telang-Cotter))*. Hence the methodology of the project was drafted with ethical responsibility taking into confidence the PPIE team’s advice. Specific ethical considerations were data management including secure data analysis and storage, privacy and security of the participants, care of informed consent, use of inclusive and culturally sensitive language, and use of social media platform nominated by the community.

## Integration of PPIE in to the research methodology

**Fig. 1:**
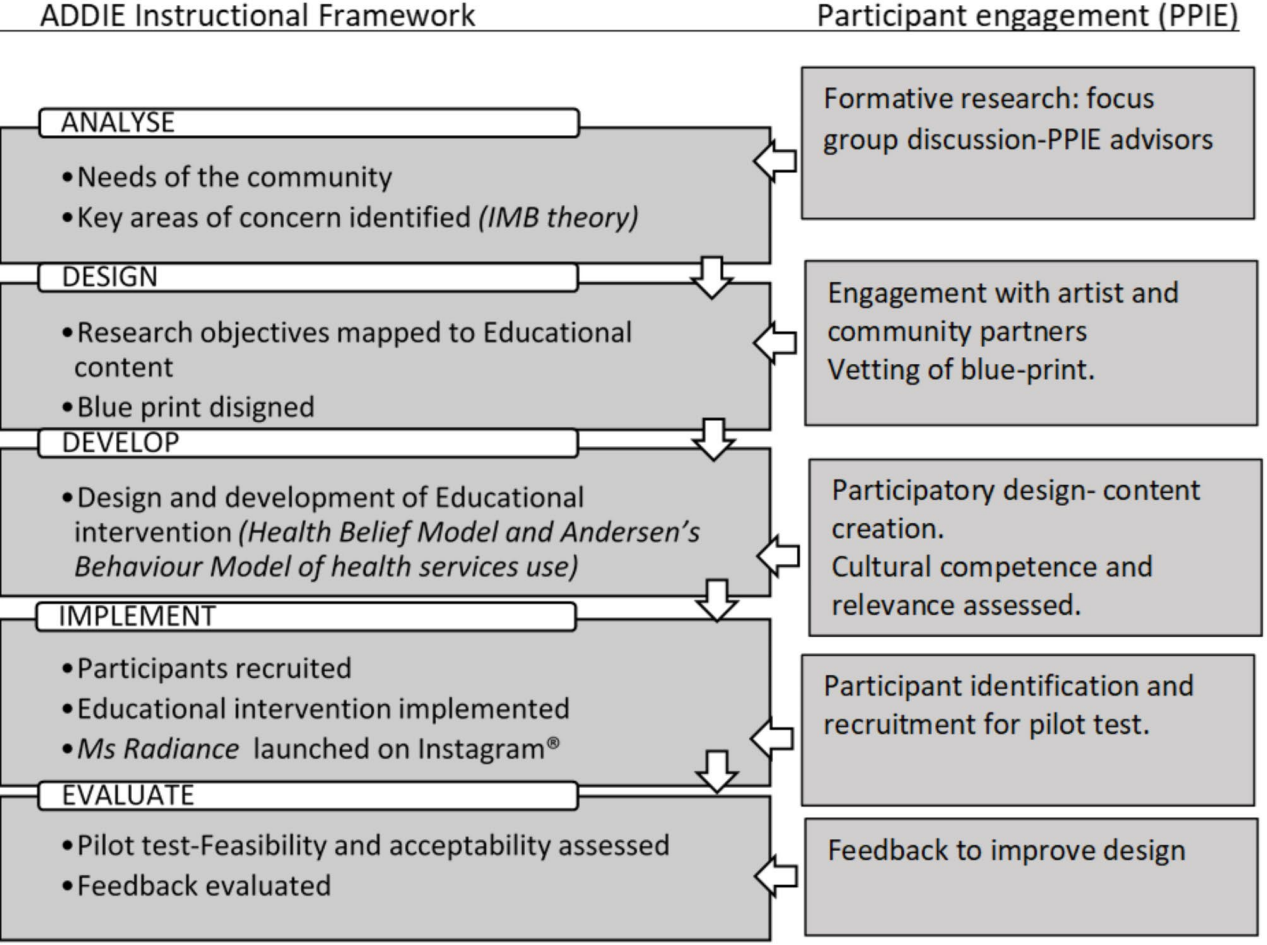
Integration of PPIE to ADDIE framework [51]

The research was part of the principal author’s (L.A.T.; a cis-gender woman) PhD project, from the School of Medicine, University College Dublin, Ireland. Facilitation of PPIE was a deliberate and gradual iterative process which took nearly two years of community engagement and trust building. To overcome inherent power imbalances in participatory research settings in marginalized communities [44–46], approaching insiders in the community to form partnerships in research was done as a preliminary step. Gaining the confidence of the community was realized through engagement in multiple informal meetings with the trans community members and advocates, attending informal gatherings, volunteering at community events and organizing community health care outreach services. A senior member in the research team (A.R.) had more experience working with the local trans community, added valuable perspectives to the PPIE approach [9, 47–49]. A more structured approach to PPIE was provided through integration of the steps in design of the project with the instructional framework of ADDIE (analyse, design, develop, implement, evaluate) [50]. (Fig. 1: Integration of PPIE to ADDIE framework) [51]. Hence the integration of PPIE approach in this project is described under the headings of [1] *analysis stage* [2], *design and development stage and* [3] *implementation and evaluation stage*.

### Integration of PPIE in the analysis stage

The concept and broad framework of the research project were prepared by the researchers in the team. A key resource person from the transgender community, an insider who had prior experience in transgender health research was entrusted with the pivotal role of research assistant (RA). Exploring the needs of the community was set as a starting point of the research, which would be analysed qualitatively. The research team initially thought that qualitative interviews would be sufficient to engage and explore the needs of the community but it became clear that a remunerated RA who was a community member was required to optimise PPIE and the project as a whole. Initial informal meetings with community members set pace of the research with both the researches and community members understanding their roles and building trust. The concept of PPIE was introduced and capacity building with collaborators was discussed. A group of transgender women, who volunteered at a local non-governmental organization (NGO) working for the transgender community agreed to participate. These women engaged in part-time sex work as a source of income and shared a unique perspective within the community that allowed them to educate their peers about access to care and safe sexual practices. The meetings were held in spaces deemed safe and accessible by the community members, for example coffee shops and community halls. These initial meetings were largely undocumented, except for handwritten notes taken by the RA. Participatory research as a concept was new to most and hence many of the community members were sceptical about their role, their ability to devote time and the goals of the project itself. After multiple sessions of brainstorming, the team consolidated the collaborators and a WhatsApp group was created with five members. The important ideas that came out of this collaboration were integrated with the formal research methodology. The research was divided into two phases: the first qualitative phase and the second intervention phase which would be analysed using mixed methods.

The first phase in the research project was conducted as in-depth interviews and focus group discussion (FDG) (results not described in this report) [47]. The aim of the qualitative research was to consolidate the needs of the community while exploring the barriers and facilitators of oral health care utilization and safe sexual practices related to oral transmission of sexually transmitted infections (STIs). The questions in the interview guide were reviewed for content and trans-friendly language and tone with the help of the PPIE collaborators. The interview participants included transgender women from the local community in and around Penang, while the FGD involved key stakeholders in transgender health care who would serve as PPIE advisors for the research. Key stakeholders in the local transgender health context are individuals, groups, or organizations that play a role in improving the health and well-being of transgender individuals [52, 53]. The qualitative analysis was guided by the Information-Motivation-Behavioural skills theory (IMB theory) [54] by addressing the informational gaps in oral health awareness and safe oral sexual practices; motivational barriers in utilizing oral health care services; and behavioural skills related to oral hygiene practices as well as safe oral sexual practices. The results of the first phase were thematically analysed and two important areas of knowledge gaps were identified. Firstly, there was insufficient oral health awareness with low oral health care utilization. Secondly and more importantly, gaps in knowledge of safe oral sexual practices were identified with most the participants perceiving a lower risk of transmission of STIs through unprotected oral sexual practices compared to anal or genital sex [47]. 

**Fig. 2:**
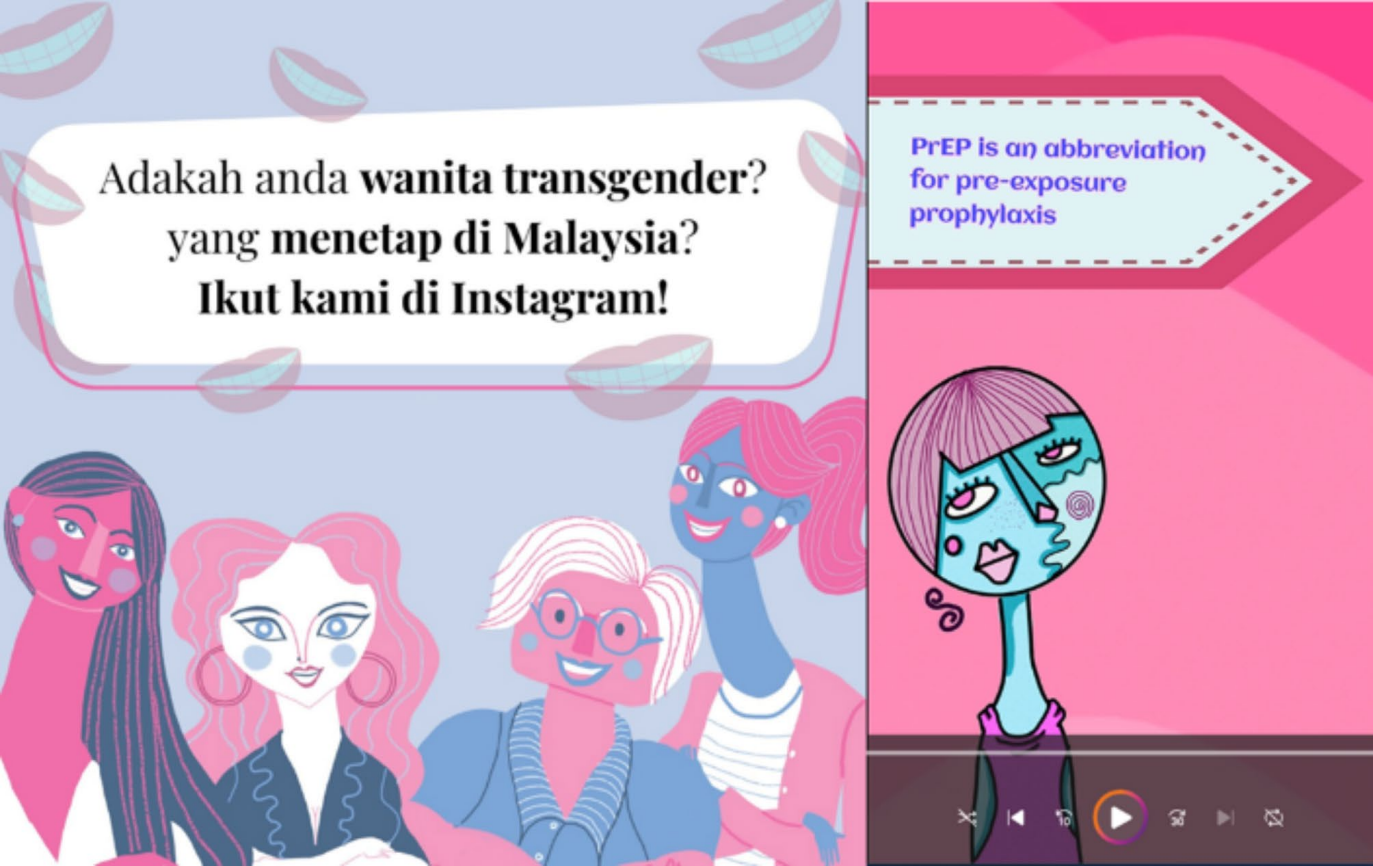
Sample artwork created for *Ms Radiance*

Addressing the gaps identified through the first phase laid the foundation for the second phase of the research. The second phase involved the design and development of an educational intervention that would be pilot tested for feasibility and acceptability and later scaled-up for a full version of the intervention. The PPIE team was approached for creating educational content that was community centred, bilingual and culturally appropriate. The use of existing social media platforms to roll out the educational content instead of a new mobile application or web-based platform was suggested by the team. Instagram^®^ was chosen as the social media platform as it was one of the most commonly visited platform at the time, with emphasis laid on visuals and many of the community members already having user profiles. An artist (Z.R.H) from the transgender community network was recruited. The role of the artist was to understand the scientific content and use art as a medium to convey the message by simplifying it and making artistically appealing at the same time. The second artist to join the team was an interview participant (B.N.) who had volunteered to contribute to the project through the unique artistic display of Cubism [36]. Cubism (Fig. 2) is a special art form that deconstructs objects into geometric shapes, presenting multiple perspectives simultaneously to challenge traditional notions of form and representation [55]. Though historically not associated with transgender expressions, the artist (B.N.) felt that Cubism as an art form resonates deeply with the themes of fluid identities experienced within the transgender community. Formal agreements between the artists and the research team were prepared and a fee for every piece of art work created was agreed upon. The artists however, decided to waiver the fee for most of the art work created as a mark of respect and commitment to betterment of the local transgender community.

The principal author and the RA served as the connecting link between the trans community and the rest of the research team. Multiple virtual meetings over zoom and google meet would follow to consolidate the research objectives and ethical approach to the research project. Setting a time frame was essential for working together as each of the collaborators had their own day jobs. Funding opportunities were explored and since PPIE is not well established as a research approach in this part of the world, these opportunities were also limited.

### Integration of PPIE in the design and development stage

The analysis stage of the project helped to consolidate the areas of focus of the educational intervention: awareness regarding oral health, oral transmission of STIs and safe oral sexual practices. The topics included in the educational intervention were prioritized and guided by the theoretical framework of the Health Belief Model [56] and Andersen’s Behaviour Model of health services use [57]. The topics were listed based on the aims of the research, existing literature and the gaps identified through first phase exploratory research with the transgender community. A blueprint was prepared with each topic and a storyboard was created with specific education content, external links and references, along with story lines and lessons learnt. The content was then translated to the local language Bahasa Malaysia by the PPIE team using colloquial and slang words wherever deemed necessary in order to give it a more relatable feel and look. The project was named *Ms Radiance* based on suggestions by the PPIE team, reflecting inner beauty and confidence, which were both important and significant concepts in the transgender community [58]. The artists created artwork based on the assigned topics in the storyboard and designed them into static images for Instagram^®^ posts, videos for reels, and stories using both of these. Avatars of four transgender women reflecting the multi-ethnic population in Malaysia were conceived (Miza, Rachel, Divya and Yin) and the interactions between them were used as storylines to deliver the message. The colour palate of the avatars was derived from in the iconic transgender flag colours. Attention to fine details was laid by the PPIE team, with an acronym of the character names adding up to the word Ms Radiance *(Miz + Ra + Di + Yin = Ms Radiance)*. The educational content with information and artwork was vetted for scientific accuracy by the experts in the research team. Posting content and manging the social media account was done by the principal author (L.A.T.). The RA and members of the PPIE team strategized the optimal time frame for each post/story, choice of music, hashtags, as well as messages that would create an impact. Figure 2 shows sample art work created for Ms Radiance.

### Integration of PPIE in the implementation and evaluation stage

*Ms Radiance* was launched on Instagram^®^ and the impact of the educational intervention was assessed with the help of a pilot study which ran for three weeks to evaluate the feasibility and acceptability of the intervention. The feasibility of the intervention was assessed by the retention rate and intervention completion rate, while the acceptability was assessed by the feedback provided by the participants and their chances of recommending the intervention to other transgender women. The recruitment of thirty participants for the pilot study through word of mouth and snow-balling sampling was spearheaded by the PPIE team. Any person identifying as a transgender woman or in the transfeminine spectrum was invited to participate. The community members and seeds from friendship networks who helped with successful recruitment were compensated for their positive research influence.

The feedback given by the participants were analysed during multiple meetings with the PPIE team. The feedback was segregated into positive feedback and scope for improvement. Feedback came through multiple channels, some participants gave written feedback through the questionnaire, while others preferred to talk with the PPIE members, who would convey the message to the research team. Feedback regarding the questionnaire was about the language used, length of the questionnaire, difficult to understand questions and options in the answers section. Feedback regarding the content on Instagram was regarding the topics, number of posts, engagement and organization of the topics. All forms of feedback from the pilot were utilised and channelised towards improvement of *Ms Radiance* for a future scaled-up version. The PPIE team worked together with the research team for over twelve months to oversee project *Ms Radiance*: the content creation, participant recruitment, data collection, gathering feedback, data analysis and preparation of a scaled-up version of the intervention. Long term sustainability of the project was planned, which would involve sharing the educational content of *Ms Radiance* with local NGOs working with transgender women: Penang Family Health Development Association (FHDA, Penang) and SEED Malaysia (Pertubuhan Pembangunan Kebajikan Dan Persekitaran Positif Malaysia) so that it can be used for educational purposes through workshops and train-the-trainer initiatives.

### Strengths of PPIE in project Ms Radiance

- Meaningful connections with the transgender community were facilitated largely though providing access and trust building.
- Contribution and involvement in artwork and content creation by making it bilingual, culturally sensitive and tailor-made to the community needs.
- Collaborative efforts in all activities related to the research project such as translation, editing of consent forms and questionnaires, participant recruitment and retention, data collection and analysis.
- Supporting the project through devoting time and continued engagement from the start to the end.

### Limitations of PPIE in project Ms Radiance

- The concept of PPIE was new to most involved in this project and hence the wider scope of PPIE may have remained less explored.
- Lack of funding allocation for PPIE meant that the project was reliant on peoples’ free time and good will and also likely participants were all in friend networks rather than more general transgender population in the community.
- Due to the ethical constrains involved in research with marginalized population, PPIE in the project could not be widely propagated, which may have affected the reach of the project.

### Recommendations

- The authors recommend exploring sustainable PPIE funding for future projects within the transgender community and other marginalized communities. Funding opportunities may be explored by integrating PPIE into existing community initiatives, collaborations with philanthropic foundations and international funding agencies as well as integrating into national health policies by ministry of health, Malaysia.
- Strategies to engage with broader community segments in Malaysia are by including ethnically, culturally and religiously diverse elements within the research. Adopting culturally tailored communication, involving community leaders, and ensuring religious and cultural sensitivity in protocols could strengthen inclusivity and improve diversity of research participants [39, 59, 60].
- Long term sustainability of educational initiatives for marginalized communities stand to benefit from partnering with youth leaders for community centric content creation, backed by funding from local sources [61].

## Conclusion

Health interventions designed for transgender populations are more likely to be purposeful when they are developed with community involvement and engagement [23–25, 30]. PPIE is achievable with the right team in the right place to include the community at every stage of the project. Through this narrative the authors have described their first-hand experiences with PPIE in designing *Ms Radiance*, a novel social media based educational intervention for improving oral health and sexual health awareness in transgender women of Malaysia. Despite limitations with funding, step wise integration of PPIE to the research methodology was achieved through trust building and continued community engagement. The lessons learnt from this project can serve as an example for empowerment of local transgender communities. The authors believe that this research fills gaps in current health literature, particularly regarding oral health and digital interventions in the global South that can also be translated to benefit other marginalized communities. Welfare organizations and NGOs can foster community ownership of similar projects and leverage the use of social media for securing funding and meaningful partnerships.

## Electronic supplementary material

Below is the link to the electronic supplementary material.

Supplementary Material 1: GRIPP-2 Checklist

## Data Availability

No datasets were generated or analysed during the current study.

## Declarations

### Reflexivity and positionality

The authors of this article include cis-gender and transgender individuals. Three of the authors are transgender women and the remaining have experience working with gender diverse populations. Five of the six authors are from the Global South, with four of them being Malaysian. All authors involved in the preparing this article ensured a reflexive approach free of potential biases.

## Abbreviations

STIs: Sexually Transmitted Infections
ADDIE: Analyse, Design, Develop, Implement and Evaluate instructional framework
PPIE: (as described by the authors in this research) Participant and Public Involvement and Engagement
IMB theory: Information-Motivation-Behavioural skills theory
