# Supplementary material for: Community involvement and engagement in designing a social-media based educational intervention for oral and sexual health promotion in transgender women in Malaysia: a narrative summary
Source: Res Involv Engagem. 2025 Mar 27;11:29. doi: 10.1186/s40900-025-00683-6 (PMC11951667; doi:10.1186/s40900-025-00683-6)
Supplement: Supplementary file 1 — Supplementary Material 1: GRIPP-2 Checklist [file 40900_2025_683_MOESM1_ESM.docx]

**Guidance for Reporting Involvement of Patients and the Public (GRIPP-2) Short form Checklist**

| **Section and topic** | **Item** | **Reported on page No** |
| --- | --- | --- |
| 1. Aim | Through this narrative the authors have described PPIE methodology with emphasis on the participation of transgender women in various aspects of the design and development of a novel digital health intervention to promote oral health and sexual health in relation to oral STIs. Their role in trust building, recruitment, content creation, delivery and feedback about the intervention is described. | 5-6 |
| 1. Methods | Hence the methodology of the project was drafted with ethical responsibility taking into confidence the PPIE team’s advice. Specific ethical considerations were data management including secure data analysis and storage, privacy and security of the participants, care of informed consent, use of inclusive and culturally sensitive language, and use of social media platform nominated by the community.  A more structured approach to PPIE was provided through integration of the steps in design of the project with the instructional framework of ADDIE (analyse, design, develop, implement, evaluate). (50) (Figure 1: Integration of PPIE to ADDIE framework). (51) Hence the integration of PPIE approach in this project is described under the headings of (1) analysis stage, (2) design and development stage and (3) implementation and evaluation stage. | 7-15 |
| 1. Study results | This is a narrative manuscript, hence there is no dedicated results and discussion section. The strengths of PPIE and limitations have however been discussed.  *Strengths of PPIE in project Ms Radiance:*   - Meaningful connections with the transgender community were facilitated largely though providing access and trust building. - Contribution and involvement in artwork and content creation by making it bilingual, culturally sensitive and tailor-made to the community needs. - Collaborative efforts in all activities related to the research project such as translation, editing of consent forms and questionnaires, participant recruitment and retention, data collection and analysis. - Supporting the project through devoting time and continued engagement from the start to the end.   *Limitations of PPIE in project Ms Radiance:*   - The concept of PPIE was new to most involved in this project and hence the wider scope of PPIE may have remained less explored. - Lack of funding allocation for PPIE meant that the project was reliant on peoples’ free time and good will and also likely participants were all in friend networks rather than more general transgender population in the community. - Due to the ethical constrains involved in research with marginalized population, PPIE in the project could not be widely propagated, which may have affected the reach of the project. | 15-16 |
| 1. Discussion and conclusions | Health interventions designed for transgender populations are more likely to be purposeful when they are developed with community involvement and engagement. (23-25, 30) PPIE is achievable with the right team in the right place to include the community at every stage of the project. Through this narrative the authors have described their first-hand experiences with PPIE in designing Ms Radiance, a novel social media based educational intervention for improving oral health and sexual health awareness in transgender women of Malaysia. Despite limitations with funding, step wise integration of PPIE to the research methodology was achieved through trust building and continued community engagement. The lessons learnt from this project can serve as an example for empowerment of local transgender communities. The authors believe that this research fills gaps in current health literature, particularly regarding oral health and digital interventions in the global South that can also be translated to benefit other marginalized communities. Welfare organizations and non-governmental organizations (NGOs) can foster community ownership of similar projects and leverage the use of social media for securing funding and meaningful partnerships. | 17-18 |
| 1. Reflections /critical perspective | Author’s perspectives:  This paper outlines the step-by-step involvement of PPIE team members, specifically individuals from the transgender community in Malaysia. The PPIE approach, relatively new to this region, marked a first-time experience for transgender women among the team members as they worked toward improving the well-being of their own community. From the initial stages of brainstorming, working alongside researchers and identifying community needs to collaboratively creating artwork, recruiting participants, and analysing data, the project was carried out as a cohesive team effort. Valuing community input at every stage significantly impacted everyone involved. Spanning nearly three years, the project not only engaged the community but also created employment opportunities, fostered social media interaction, sparked interest, and raised awareness—all made possible through the active involvement and engagement of the community.  Reflexivity and positionality statement:  The authors of this article include cis-gender and transgender individuals. Three of the authors are transgender women and the remaining have experience working with gender diverse populations. Five of the six authors are from the Global South, with four of them being Malaysian. All authors involved in the preparing this article ensured a reflexive approach free of potential biases. | 21 |
